# Prunetin ameliorates myocardial pyroptosis after myocardial infarction by inhibiting the TNF-α/p38 MAPK/ERK pathway

**DOI:** 10.3389/fcvm.2026.1847407

**Published:** 2026-07-28

**Authors:** Huimin Wu, Hui Zhang, Xinyue Ding, Min Li, Daying Wang, Junqing Gao, Rui Wang, Zhen Qi, Xuan Zhao, Zongjun Liu

**Affiliations:** 1 Shanghai Putuo Central School of Clinical Medicine, Anhui Medical University, Shanghai, China; 2 The Fifth Clinical Medical College, Anhui Medical University, Anhui, China; 3 Department of Cardiology, Putuo Hospital, Shanghai University of Traditional Chinese Medicine, Shanghai, China; 4 Neocellmed Co., Ltd., Shanghai, China

**Keywords:** inflammatory response, myocardial infarction, prunetin, pyroptosis, TNF-α

## Abstract

**Background:**

The inflammatory response in cardiac tissue after myocardial infarction (MI) plays a critical role in myocardial injury. Prunetin (PRU), a natural flavonoid compound, has notable anti-inflammatory properties; however, its role in post-MI myocardial injury and the underlying molecular mechanisms remain unclear.

**Methods:**

Network pharmacology was used to predict potential targets of PRU in MI, identifying TNF-α as a high-affinity target. This interaction was further validated by molecular dynamics (MD) simulation and surface plasmon resonance (SPR) assays. A mouse MI model was established to evaluate the protective effects of PRU using echocardiography, histological staining, and serological assays. RNA sequencing was performed to explore the underlying mechanisms, and Western blotting and immunofluorescence were used to assess the TNF-α/p38 MAPK/ERK signaling pathway and pyroptosis-related proteins. R848, a TLR7/8 agonist, was used to induce TNF-α expression and evaluate whether TNF-α activation could reverse the protective effects of PRU.

**Results:**

*In vivo* and *in vitro* experiments showed that PRU significantly improved cardiac function in MI mice, reduced serum levels of myocardial injury markers, attenuated myocardial fibrosis, and decreased inflammatory cytokine release. PRU also markedly downregulated pyroptosis-related proteins, including NLRP3 and GSDMD-N. Mechanistically, PRU inhibited MI-induced activation of the TNF-α/p38 MAPK/ERK signaling pathway, thereby suppressing cardiomyocyte pyroptosis. R848 reversed the cardioprotective effects of PRU. These findings were further validated in models of damage to cardiac organoids and cardiomyocytes induced by glucose-oxygen deprivation (OGD). Notably, the data on PRU's protective effects in human cardiac organoid models were highly consistent with observations in mice, further reinforcing the clinical relevance and translational potential of this mechanistic hypothesis.

**Conclusions:**

PRU attenuates post-MI myocardial injury by inhibiting TNF-α/p38 MAPK/ERK signaling, thereby reducing cardiomyocyte pyroptosis and inflammatory responses. These findings provide a theoretical basis for the potential development of PRU as a therapeutic candidate for post-MI myocardial injury.

## 1 Introduction

MI is a life-threatening coronary event and one of the leading causes of death worldwide (1, 2). Current clinical strategies mainly focus on rapid revascularization of occluded arteries, plaque stabilization, and thrombosis prevention. However, limiting excessive post-infarction inflammation while preserving myocardial viability remains a major clinical challenge. In this context, identifying plant-derived bioactive compounds with immunomodulatory properties may offer promising therapeutic opportunities to improve outcomes after MI.

Myocardial ischemia and hypoxia disrupt local microenvironmental homeostasis and trigger inflammatory responses characterized by cytokine release within cardiac tissue (3). Excessive inflammation is a key driver of cardiac injury after MI (4–6). TNF-α, a well-established pro-inflammatory cytokine (7), binds to its receptor TNFR and activates downstream p38 MAPK and ERK signaling cascades, thereby regulating cell death and inflammatory responses (8–12).

Excessive inflammation can also activate the NLRP3 inflammasome, leading to pyroptosis, a form of programmed necrotic cell death closely associated with inflammation (13). As a key sensor of cellular damage, NLRP3 promotes caspase-1 activation, gasdermin D (GSDMD) cleavage, and the maturation and release of IL-1β and IL-18, thereby amplifying inflammatory signaling, inducing cardiomyocyte pyroptosis, and aggravating cardiac dysfunction (14, 15). Pyroptosis is executed by the N-terminal fragment of GSDMD (GSDMD-N), which disrupts plasma membrane integrity, resulting in cell lysis and the release of pro-inflammatory mediators (16, 17). Accumulating evidence indicates that cardiomyocyte pyroptosis is a critical pathological process in myocardial ischemia-reperfusion injury (18). Therefore, targeting TNF-α-mediated inflammatory signaling and downstream pyroptosis may be a promising strategy for alleviating myocardial injury after MI.

Against this backdrop, natural small-molecule agents are regarded as a promising adjunctive therapeutic strategy given their multi-targeting properties, low toxicity, and favorable safety profiles. Nevertheless, there is a critical scarcity of such molecules with well-characterized anti-pyroptotic effects and elucidated mechanisms of action. Accordingly, screening medicinal plants for immunomodulatory bioactive constituents represents a viable approach for ameliorating post-infarction cardiac outcomes.

PRU, a naturally occurring isoflavone found in dietary sources such as almonds and cherries, has been reported to possess anti-inflammatory, antioxidant, and lipid-regulating activities (19–21). Specifically, PRU has demonstrated significant anti-inflammatory protective effects in inflammatory diseases of the gut and nasal cavity (20, 21). Nevertheless, the cardioprotective role of PRU in post-MI injury and its underlying molecular mechanisms remain unclear.

In this study, we first predicted PRU-related targets and then validated TNF-α as a key target. We then systematically investigated the cardioprotective effects of PRU against ischemia/hypoxia-induced myocardial injury using an *in vivo* murine MI model and an *in vitro* cardiac organoid model subjected to OGD. Through echocardiography, molecular biology, and histological analyses, we explored whether PRU protects against MI by modulating the TNF-α/p38 MAPK/ERK signaling pathway and suppressing inflammation and cardiomyocyte pyroptosis. These findings may provide a theoretical basis for the potential application of PRU in the clinical management of MI.

## 2 Materials and methods

### 2.1 Reagents and materials

Prunetin (PRU; HPLC ≥97%, Cat. No. B30453) and resiquimod (R848; Cat. No. S80363) were purchased from Yuanye Bio-Technology Co., Ltd. (China). Recombinant human TNF-α protein was obtained from TargetMol (Cat. No. TMPK-00120, USA). Bovine serum albumin, DMEM, and the CCK-8 kit were obtained from Yuanjian Biotech (Cat. No. BSAS100, CV22001, and T4CCK1000, China). The BCA protein assay kit was purchased from Beyotime (Cat. No. P0010, China). Assay kits for superoxide dismutase (SOD), malondialdehyde (MDA), and lactate dehydrogenase (LDH) were purchased from Nanjing Jiancheng Institute (Cat. No. A001-3-2, A003-1-2, and A020-2-2, China). Mouse NT-proBNP ELISA kits were obtained from Tongwei Biotech (Cat. No. TW9810, China), as were human NT-proBNP, TNF-α, and IL-1β ELISA kits (Cat. No. TW848, TW4070, and TW15343, China). The protein extraction kit was purchased from Solarbio (Cat. No. BC3710, China). Trypsin-EDTA (0.25%) was obtained from Thermo Fisher Scientific (Cat. No. 25200072, USA). Dimethyl sulfoxide (DMSO) was obtained from Boster (Cat. No. 67-68-5, China), and glucose-free DMEM was purchased from Gibco (Cat. No. 11966025, USA). CHIR99021, BMP4, LY294002, Activin A, IWR1, retinoic acid, and FGF2 were purchased from MCE (Cat. No. HY-10182, HY-P7007, HY-10108, HY-P70311, HY-12238, HY-14649, and HY-P7004, USA). Insulin was obtained from Pricella (Cat. No. PB180432, China). The following primary antibodies were used: anti-TNF-α (Cat. No. ab307164; Abcam, UK); anti-p38 and anti-ERK1/2 (Cat. No. AF7668 and AF1051; Beyotime, China); anti-phospho-p38, anti-phospho-ERK1/2, and anti-β-tubulin (Cat. No. 9216S, 4695T, and 2128S; Cell Signaling Technology, USA); anti-GAPDH (Cat. No. ab181602; Abcam, USA); anti-Gasdermin D/Cleaved N-terminal GSDMD (Cat. No. PU224937S; Abmart, China); anti-NLRP3 (Cat. No. P60622R3S; Abmart, China); and anti-IL-1β (Cat. No. K0099661P; Solarbio, China). ECL substrate was purchased from Bio-Rad (Cat. No. 1705061, USA). The PrimeScript™ RT Reagent Kit was obtained from TaKaRa (Cat. No. RR047A, China).

### 2.2 Network pharmacology

Potential targets of PRU were retrieved from the HERB (High-Throughput Experiment- and Reference-Guided Database of Traditional Chinese Medicine), ITCM (Integrated Traditional Chinese Medicine Database), ETCM (The Encyclopedia of Traditional Chinese Medicine), and SwissTargetPrediction databases. MI-associated targets were obtained from the GeneCards database (https://www.genecards.org) using the search term “myocardial infarction”.

### 2.3 GO and KEGG pathway enrichment analysis

The STRING database (https://string-db.org/) was used to construct a protein-protein interaction (PPI) network for the potential targets of PRU in MI. Cytoscape software was then used to visualize the PPI network, and the top 10 targets with the highest betweenness scores were selected as core targets. Gene Ontology (GO) and Kyoto Encyclopedia of Genes and Genomes (KEGG) enrichment analyses of these core targets were performed using the DAVID database to identify key biological processes and signaling pathways.

### 2.4 Molecular docking validation

Protein structures were obtained from the Protein Data Bank and prepared using PyMOL by removing water and solvent molecules. Hydrogen atoms were added using AutoDock Tools, and each protein was defined as a receptor and exported in PDBQT format. The PRU structure in MOL2 format was obtained from the TCMSP database and prepared as a ligand using AutoDock Tools. Molecular docking was performed with AutoDock, and the conformation with the strongest binding affinity was selected. Docking results were visualized using PyMOL.

### 2.5 Molecular dynamics (MD) simulation

To evaluate the stability of the TNF-α-PRU interaction, molecular dynamics simulations were performed using the AMBER99SB force field and the SPC water model. The simulation temperature was set to 300 K, and the simulation time was 100 ns. Energy minimization was performed using the steepest descent method, followed by equilibration to stabilize the system.

### 2.6 Surface plasmon resonance (SPR) analysis

SPR was performed to evaluate the binding affinity between TNF-α and PRU. Recombinant human TNF-α protein was diluted to 20 μg/mL and immobilized on the FC2 channel at a flow rate of 10 μL/min, while the FC1 channel was used as a reference without immobilized ligand. The chip was blocked with 1 mol/L ethanolamine hydrochloride. PRU was serially diluted to six concentrations ranging from 0.1 to 3.13 μM. The analytes were injected sequentially from low to high concentration into both FC1 and FC2 channels at a flow rate of 20 μL/min. The association and dissociation phases were set to 100 s and 180 s, respectively. Calculate the binding rate constant (ka), the dissociation rate constant (kd), and the equilibrium dissociation constant (KD = kd/ka). The equilibrium dissociation constant KD reflects the binding strength between a small molecule and a target protein: the smaller the KD value, the higher the affinity. It is typically defined as follows: 1 nM ≤ KD < 1 μM is considered high affinity, 1 μM ≤ KD < 10 μM is considered moderate affinity, and KD ≥ 10 μM is considered low affinity.

### 2.7 RNA-Seq data analysis

Total RNA was sequenced on the Illumina platform. Raw reads were quality-filtered using fastp, and clean reads were aligned to the reference genome using STAR. Gene expression levels were quantified using featureCounts. Differentially expressed genes (DEGs) were identified using DESeq2 with thresholds of *P* < 0.05 and |log_2_FC| > 1. GO and KEGG enrichment analyses were then performed to annotate functions.

### 2.8 Animals

Male C57BL/6J mice, aged 8 weeks and weighing 20–22 g, were purchased from Beijing SPF Biotechnology Co., Ltd. All animal experiments were conducted in accordance with the National Institutes of Health Guide for the Care and Use of Laboratory Animals and were approved by the Ethics Committee of Putuo Central Hospital of Shanghai (No. DWEC-A-2025-20-1-118). Mice were housed under standard conditions and acclimated for 1 week before experimentation. Mice were anesthetized by intraperitoneal injection of 1% sodium pentobarbital and connected to a small-animal ventilator for respiratory support. Thoracotomy was performed to expose the heart, followed by ligation of the left anterior descending (LAD) coronary artery. Successful MI modeling was confirmed by the immediate appearance of a pale region on the ventricular wall after ligation. Mice in the sham group underwent the same surgical procedure without LAD ligation.

### 2.9 Grouping and treatment

After MI modeling, a total of 4 mice were excluded due to death, failure to achieve LVEF criteria (LVEF < 50%), or poor overall condition. No experimental animals died during the subsequent experimental procedures. Mice that survived 48 h post-MI underwent echocardiographic assessment. Those with comparable LVEF values meeting the inclusion criteria were handed over to a researcher not involved in this study and assigned to each experimental group using a simple randomization method, with 5 mice per group: sham, MI, PRU, PRU + R848, and enalapril (ENA) positive control group. The PRU dose was selected based on previous studies (20, 21). PRU and R848 were dissolved in DMSO to prepare stock solutions at 12 mg/mL and 75 mg/mL, respectively, and diluted to the appropriate concentrations before administration, based on body weight. ENA was freshly prepared in saline at 2 mg/kg/day. Mice in the PRU group received PRU by oral gavage at 20 or 40 μg/kg/day in a volume of 100 μL for 28 consecutive days. Mice in the PRU + R848 group received the same PRU regimen combined with intraperitoneal injection of R848 at 2 mg/kg in a volume of 100 μL twice weekly. Mice in the sham and MI groups received oral gavage with saline containing an equivalent amount of DMSO. All groups except the PRU + R848 group received intraperitoneal injections of saline containing an equivalent amount of DMSO. At the end of the experiment, cardiac tissues and serum samples were collected and stored at −80 °C.

### 2.10 Echocardiography

Echocardiography was performed in a single-blind manner. Chest hair was removed before the procedure, and mice were anesthetized with 2% isoflurane inhalation. Cardiac function was assessed using a high-resolution small-animal ultrasound imaging system equipped with a dedicated high-frequency linear array probe. The probe was gently placed on the precordial region, and the angle was adjusted to obtain M-mode ultrasound images.

### 2.11 TTC staining

After MI, mouse hearts were excised and cut longitudinally into 2-mm-thick sections. The sections were fully immersed in 2% TTC solution and incubated at 37 °C for 15 min. The slices were then fixed in paraformaldehyde. Red staining indicated viable myocardial tissue, whereas white areas represented infarcted tissue.

### 2.12 Histopathological analysis

Histopathological analysis was performed in a single-blind manner. Fresh cardiac tissues were fixed in 4% paraformaldehyde for 24 h, dehydrated through a graded ethanol series, embedded in paraffin, and sectioned. The sections were stained with hematoxylin and eosin (H&E) and Masson's trichrome. H&E staining was used to assess myocardial morphology, cardiomyocyte arrangement, and inflammatory infiltration. In Masson-stained sections, collagen fibers appeared blue, and cardiomyocytes appeared red. The fibrotic area was quantified using ImageJ software.

### 2.13 ELISA

Samples were thawed on ice before analysis. Serum NT-proBNP levels in mice and TNF-α and IL-1β levels in cardiac organoid culture supernatants were measured using ELISA kits according to the manufacturer's instructions.

### 2.14 Cardiac organoid model

The research team collaborated with Cellapy (Beijing, China) to isolate and generate iPSCs from human peripheral blood; this study has been approved by the Ethics Committee of Putuo Hospital Affiliated to Shanghai University of Traditional Chinese Medicine [No. PTEC-A-2022-13(S)-1]. The iPSCs were routinely thawed, passaged, and expanded. When the cells reached approximately 90% confluence, cardiac organoid differentiation was initiated according to established protocols in our laboratory (22–24). Briefly, cells were dissociated and seeded into 96-well plates at approximately 9,000 cells per well in 100 µL of mTeSR medium. After 24 h, the medium was replaced with 100 µL of differentiation medium A containing LY294002, Activin A, CHIR99021, BMP4, and insulin. After 48 h, the medium was replaced with differentiation medium B containing IWR1, BMP4, FGF2, retinoic acid, and insulin, which was maintained for 4 days. The medium was then replaced with differentiation medium C containing FGF2, and insulin was added daily for 5 days. Finally, organoids were cultured in insulin-supplemented maintenance medium, which was refreshed daily. For hypoxic treatment, mature cardiac organoids were transferred to glucose- and serum-free DMEM and cultured in a tri-gas incubator containing 5% O_2_ for 24 h.

### 2.15 Cell culture

The H9C2 rat cardiomyocyte cell line (GNR-5) was purchased from the Shanghai Cell Bank, Chinese Academy of Sciences. Cells were cultured in DMEM supplemented with 10% fetal bovine serum and 1% penicillin-streptomycin at 37 °C in a humidified incubator with 5% CO_2_. Cells were passaged when confluence reached approximately 80%, and subsequent experiments were performed using cells in good condition.

### 2.16 Cell grouping and drug administration

H9C2 cells were seeded in microplates and randomly assigned to the control, OGD, and PRU-treated groups at different concentrations. After cell attachment, cells in the control group were maintained in normal medium, whereas cells in the OGD and PRU groups were cultured in glucose- and serum-free medium. PRU was added to the corresponding treatment groups. Cells in the OGD and PRU groups were incubated at 37 °C under 5% CO_2_ and 1% O₂ for 6 h.

### 2.17 Cell viability assay

H9C2 cells were seeded in 96-well plates. When cell confluence reached 60%–70%, cells were treated with serial concentrations of PRU: 0, 2, 4, 6, 8, 10, and 20 μM for 24 h. After treatment, 10 μL of CCK-8 solution was added to each well, followed by incubation for 2 h. Absorbance at 450 nm was measured using a microplate reader.

### 2.18 Determination of LDH, SOD, and MDA levels

Commercial kits were used to measure LDH, SOD, and MDA levels as indicators of cellular injury and oxidative stress. Cell culture supernatants were collected and processed according to the manufacturer's protocols. Absorbance was measured at the specified wavelengths using a microplate reader, and final concentrations were calculated according to the formulas provided by the kit manufacturers.

### 2.19 qPCR analysis

Total RNA was extracted from cardiac tissues using TRIzol reagent, and cDNA was synthesized using the PrimeScript™ RT Reagent Kit. qPCR was performed using TB Green® Premix Ex Taq™. Relative gene expression was calculated using the 2^−ΔΔCt^ method. Primer sequences are listed in Table 1.

**Table 1:** Primer sequences.

Genes (Rat)	Primer sequences	
18s	Forward	5′-TCAAGAACGAAAGTCGGAG-3′
	Reverse	5′-GGACATCTAAGGGCATCAC-3′
TNF-α	Forward	5′-CGGAAAGCATGATCCGAGAT-3′
	Reverse	5′-AGACAGAAGAGCGTGGTGGC-3′
EGFR	Forward	5′-CCAAGGCACAAGTAACAGGC-3′
	Reverse	5′-TTCCAAGTTTCCAAGGACCAC-3′
AKT1	Forward	5′-CCTGGACTACTTGCACTCCG-3′
	Reverse	5′-CACAGCCCGAAGTCCGTTAT-3′
PPARG	Forward	5′-AGCTCTGTGGACCTCTCTGT-3′
	Reverse	5′-GACGCAGGGGCGGAATAAT-3′
ACTB	Forward	5′-GGACCTGACAGACTACCTCA-3′
	Reverse	5′-GTTGCCAATAGTGATGACCT-3′

Genes (Mice)	Primer sequences	
TNF-α	Forward	5′-AAGGCCGGGGTGTCCTGGAG-3
	Reverse	5′-AGGCCAGGTGGGGACAGCTC-3′
IL-6	Forward	5′-GACTGATGCTGGTGACAAC-3′
	Reverse	5′-GACAGGTCTGTTGGGAGTG-3′
IL-1β	Forward	5′-GAAATGCCACCTTTTGACAGT-3′
	Reverse	5′-TGGATGCTCTCATCAGGACAG-3′

### 2.20 Western blotting

After isolation, cardiac tissues were immediately cut into approximately 30-mg pieces and lysed in pre-chilled RIPA buffer on ice for 10 min. The samples were homogenized using a tissue disruptor for three cycles of 30 s each, with 30-s intervals. After centrifugation, the supernatant was collected. Total protein was separated by SDS-PAGE and transferred onto PVDF membranes. The membranes were blocked with 5% nonfat milk in TBST for 2 h, incubated with primary antibodies overnight at 4 °C, washed, and then incubated with secondary antibodies at room temperature for 2 h. Protein bands were visualized using ECL reagent and quantified using ImageJ software.

### 2.21 Statistical analysis

All experimental data were analyzed statistically using GraphPad Prism 8.0 software. For data that conform to a normal distribution and have homogeneous variance. Comparisons between the two groups were using Student's *t*-test; whereas Welch's *t*-test was used for data with unequal variances. For multiple comparisons, one-way ANOVA followed by Tukey's *post hoc* test was performed for normally distributed data with equal variances; otherwise, the Kruskal–Wallis test with Dunn's *post hoc* test was used. All data are presented as mean ± SD, and *P* < 0.05 was considered statistically significant.

## 3 Results

### 3.1 PRU ameliorates cardiac dysfunction and fibrosis in mice after MI

**Figure 1:**
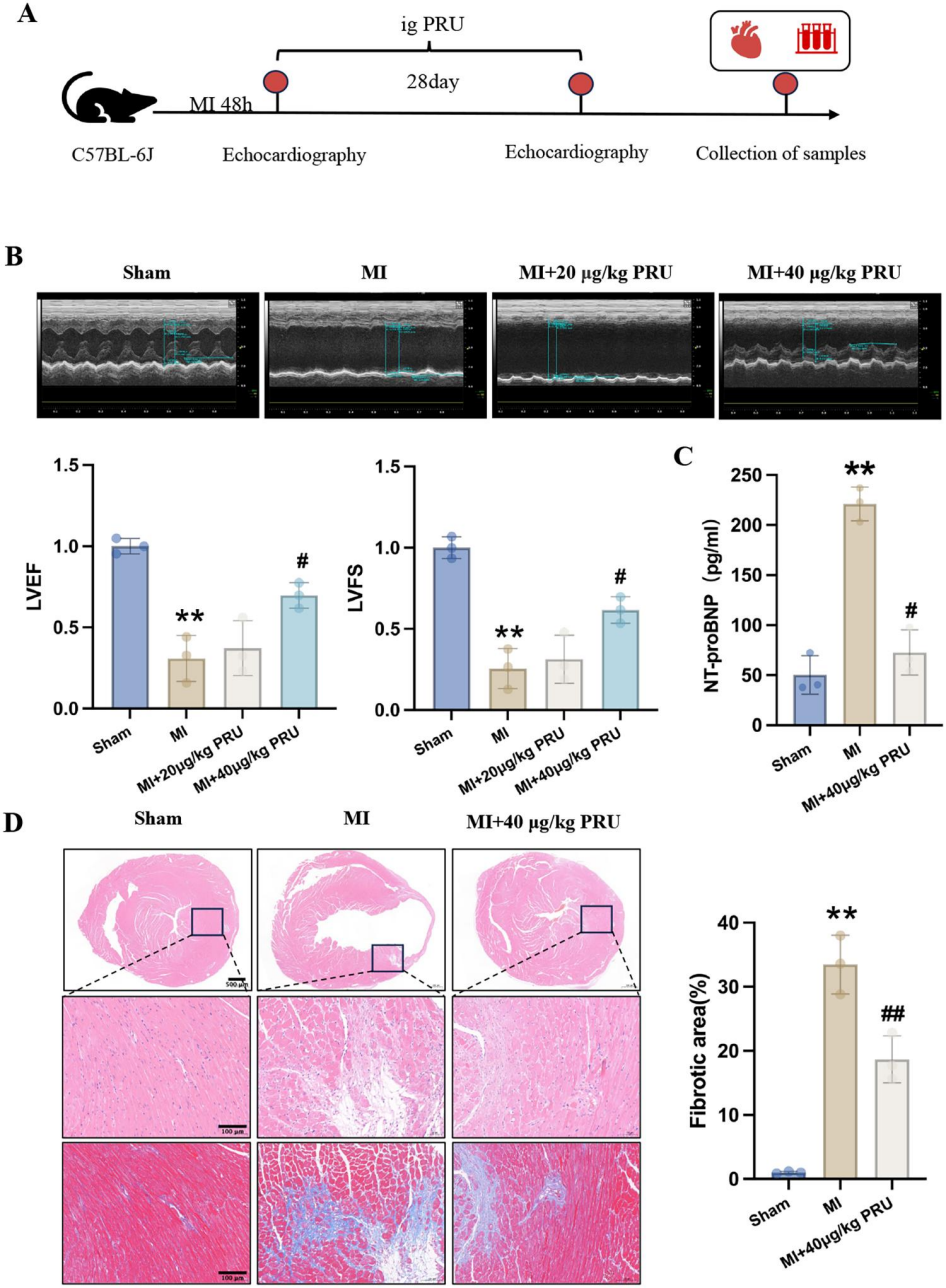
PRU ameliorates cardiac function and fibrosis in mice with MI. **(A)** Experimental flowchart of mouse experiments. **(B)** Representative echocardiographic images of each group and statistical graphs of LVEF and LVFS, *n* = 3. **(C)** Quantitative analysis of serum NT-proBNP levels, *n* = 3. **(D)** Representative images of heart sections stained with HE and Masson, and quantitative analysis of cardiac fibrosis area (%), scale bar = 500 µm (upper), scale bar = 100 µm (lower), *n* = 3. Data are presented as mean ± SD from independent biological replicates. For panels **(B–D)**, which passed the normality assumption, one-way ANOVA followed by Tukey's *post-hoc* test was applied. Compared with the Sham group, **P < 0.01, *P < 0.05; compared with the MI group, **^##^***P* < 0.01, **^#^***P* < 0.05; compared with the MI + PRU group.

To establish the MI model, mice underwent LAD ligation, and obvious infarct lesions were confirmed by TTC staining 48 h after surgery (Supplementary Figure S1B). After 28 consecutive days of PRU treatment, cardiac function was evaluated by echocardiography (Figure 1A). PRU treatment improved cardiac function in post-MI mice, as evidenced by significant increases in LVEF and LVFS, along with reduced serum NT-proBNP levels (Figures 1B,C). Consistently, Masson's trichrome and H&E staining showed that PRU markedly attenuated MI-induced myocardial fibrosis and pathological injury (Figure 1D). Together, these results indicate that PRU, particularly at 40 μg/kg, significantly ameliorates cardiac dysfunction and suppresses myocardial fibrosis progression in mice after MI.

### 3.2 Network pharmacology combined with multi-method validation identifies potential targets of PRU in post-MI myocardial injury

**Figure 2:**
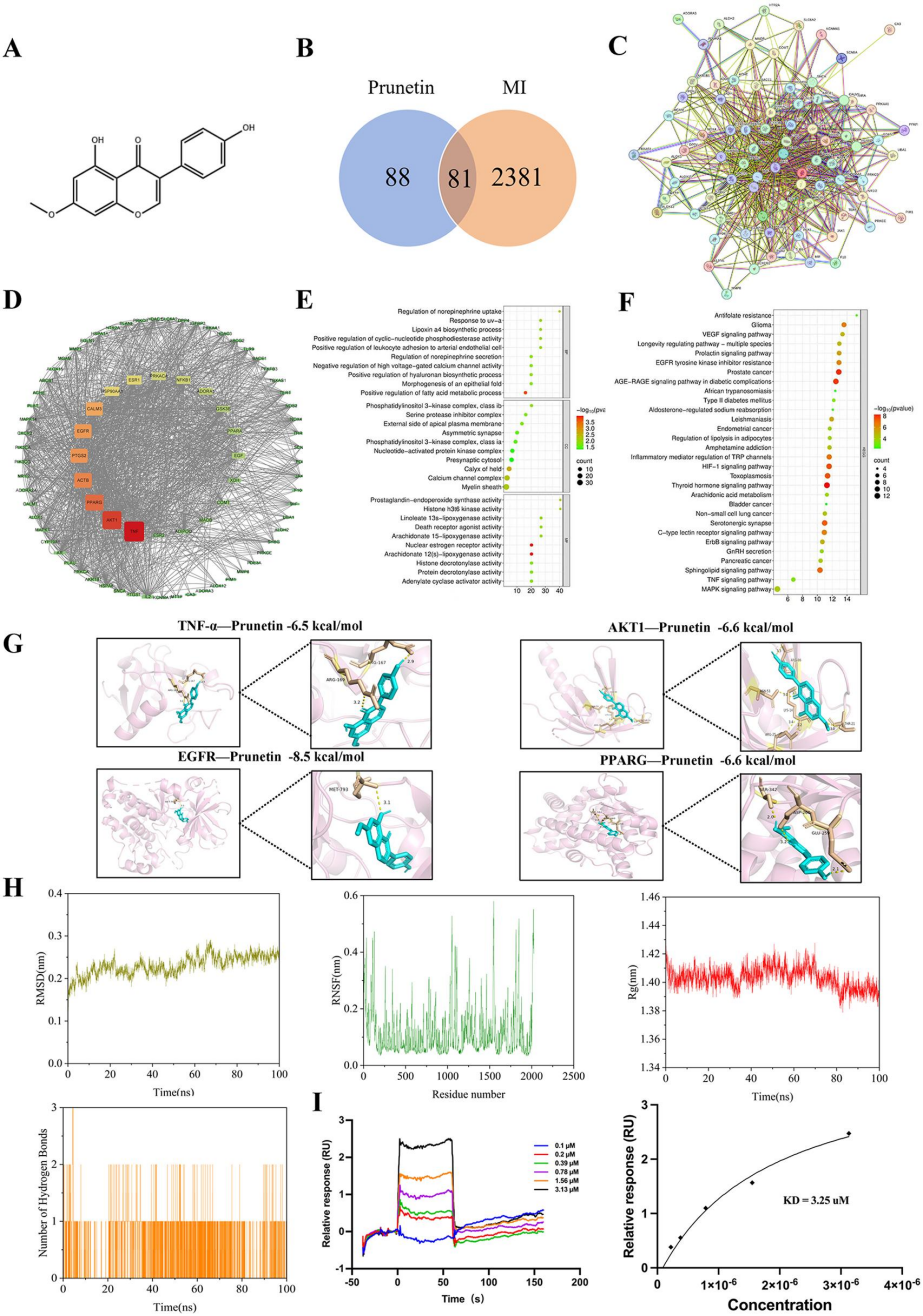
Multi-technology screening and verification of potential targets of PRU for MI therapy. **(A)** Chemical structure of PRU. **(B)** Venn diagram of PRU targets and MI-related targets. **(C)** PPI network. **(D)** Visualization of core gene PPI network. **(E)** GO functional enrichment analysis. **(F)** KEGG pathway enrichment analysis. **(G)** Molecular docking of PRU with targets. **(H)** MD simulation of PRU and TNF-α. **(I)** SPR assay for binding affinity between PRU and TNF-α.

We next used a multi-technology strategy to identify potential therapeutic targets underlying the cardioprotective effects of PRU after MI. The molecular structure of PRU was first obtained from the MolView website (Figure 2A). A total of 169 predicted PRU-related targets and 2,462 MI-associated targets were retrieved from public databases, among which 81 overlapping targets were identified by intersection analysis (Figure 2B). Based on these common targets, a PPI network was constructed, and the top 10 hub targets, including TNF-α, AKT1, PPARG, and ACTB, were identified (Figures 2C,D). GO enrichment analysis was then performed to characterize the biological functions of the overlapping targets. KEGG pathway analysis revealed that these targets were primarily enriched in signaling pathways closely associated with cellular stress responses and inflammation regulation, including the C-type lectin receptor, HIF-1, MAPK, and TNF signaling pathways (Figures 2E,F). Given that MAPK serves as a key downstream signaling branch of the TNF pathway and plays a crucial role in regulating the expression of inflammatory cytokines and pyroptosis, we selected the MAPK pathway for further validation. Molecular docking further suggested that PRU exhibited strong binding affinity with the identified hub targets (Figure 2G). To further prioritize candidate targets, qPCR screening was performed in the OGD-induced cardiomyocyte injury model. Among the hub targets, PRU significantly downregulated TNF-α mRNA expression (Supplementary Figure S1A). MD simulation and SPR analysis further confirmed a stable, moderate affinity interaction between PRU and TNF-α (Figures 2H,I).

### 3.3 PRU improves post-MI cardiac dysfunction by targeting TNF-α

**Figure 3:**
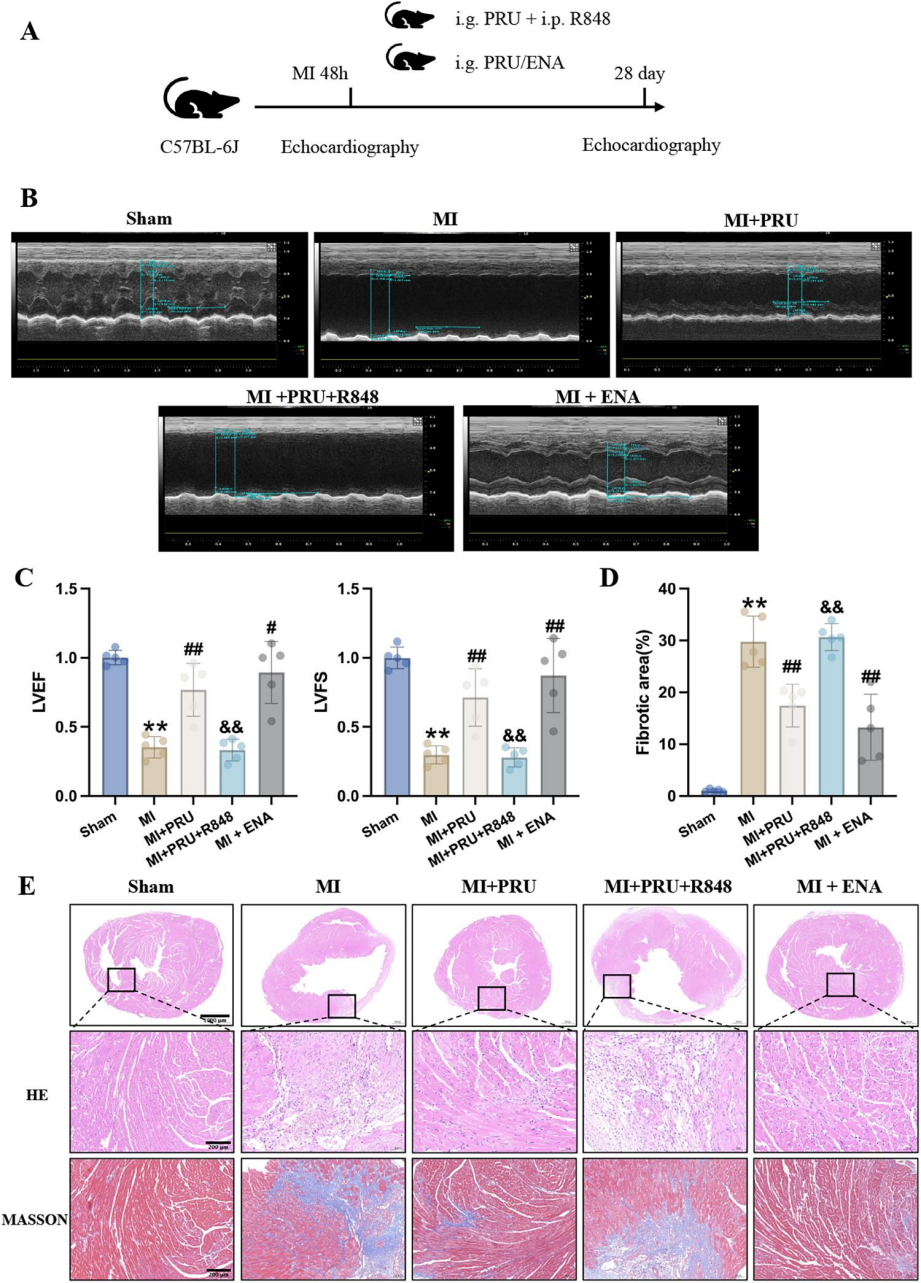
PRU improves cardiac function in MI mice by inhibiting TNF-α function. **(A)** Schematic flowchart of *in vivo* experiments. **(B)** Representative echocardiographic images from each experimental group. **(C)** Quantitative analysis of LVEF and LVFS, *n* = 5. **(D,E)** HE and Masson staining show representative images of cardiac tissue and quantitative analysis of cardiac fibrosis area (%), scale bar = 1,000 µm (upper), scale bar = 200 µm (lower), *n* = 5. Data are presented as mean ± SD from independent biological replicates. For panels **(B–D)**, which passed the normality assumption, one-way ANOVA followed by Tukey's *post-hoc* test was applied. Compared with the Sham group, **P < 0.01, *P < 0.05; compared with the MI group, **^##^***P* < 0.01, **^#^***P* < 0.05; compared with the MI + PRU group, **^&&^***P* < 0.01, **^&^***P* < 0.05.

To further verify whether TNF-α serves as a key target of PRU against myocardial injury *in vivo*, MI mice were treated with PRU alone or in combination with R848, an inducer of TNF-α expression. An ENA group was included as a positive control. The experimental design is shown in Figure 3A. At 48 h after MI, the LVEF and LVFS of mice in the surgery group were significantly lower than those in the Sham group, and there was no significant difference in these two indicators between the surgery groups (Supplementary Figure S1C). After the corresponding interventions, PRU markedly improved MI-induced cardiac dysfunction, as evidenced by increased LVEF and LVFS (Figures 3B,C). PRU also attenuated myocardial fibrosis and inflammatory infiltration (Figures 3D,E). However, co-treatment with R848 significantly weakened these cardioprotective effects.

**Figure 4:**
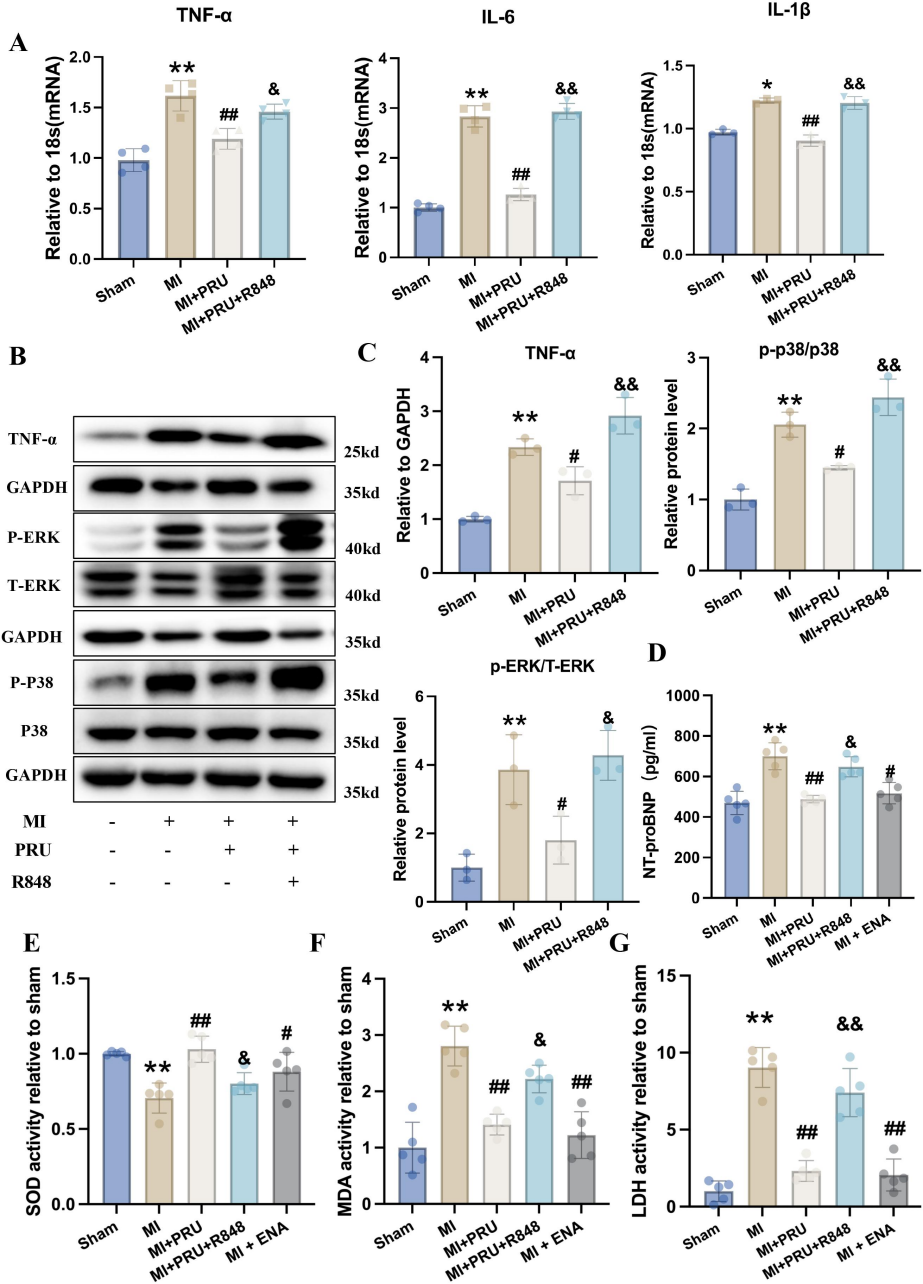
Effects of PRU on TNF-α pathway proteins and oxidative stress markers in infarcted myocardium. **(A)** mRNA expression levels of TNF-α, IL-6, and IL-1β in cardiac tissue, *n* = 3. **(B,C)** Expression of TNF-α, p-p38, and p-ERK proteins in cardiac tissue measured and quantified by WB, *n* = 3. **(D)** Serum NT-proBNP levels in MI mice, *n* = 5. **(E–G)** Levels of SOD, MDA, and LDH in mice cardiac tissue, *n* = 5. Data are presented as mean ± SD from independent biological replicates. For panels **(A,C,D,F,G)**, which passed the normality assumption, one-way ANOVA followed by Tukey's *post-hoc* test was applied. For panel **(E)**, which violated the normality assumption, the Kruskal–Wallis test followed by Dunn's *post-hoc* test was used for multiple comparisons. Compared with the Sham group, **P < 0.01, *P < 0.05; compared with the MI group, **^##^***P* < 0.01, **^#^***P* < 0.05; compared with the MI + PRU group, **^&&^***P* < 0.01, **^&^***P* < 0.05.

Consistently, PRU significantly reduced the mRNA levels of TNF-α, IL-1β, and IL-6 in cardiac tissues after MI (Figure 4A). PRU also decreased the protein levels of TNF-α, phosphorylated p38, and phosphorylated ERK in cardiac tissues (Figures 4B,C), whereas R848 markedly reversed these effects. These results suggest that activation of the TNF-α/p38 MAPK/ERK signaling pathway promotes inflammatory cytokine release and aggravates post-MI myocardial injury, whereas PRU suppresses this pathway, thereby alleviating inflammation and cardiac damage. In addition, PRU significantly decreased serum NT-proBNP levels in MI mice, and this effect was also reversed by R848 (Figure 4D). Analysis of oxidative stress and cellular injury markers showed that PRU reduced LDH activity and MDA levels while increasing SOD activity in cardiac tissues. These regulatory effects were similarly abolished by R848 (Figures 4E,G). Moreover, PRU also inhibited this pathway and alleviated OGD-induced injury in H9C2 cardiomyocytes, whereas R848 reversed these protective effects (Supplementary Figure S2). Collectively, these findings indicate that TNF-α is a key target through which PRU mitigates post-MI myocardial injury.

### 3.4 PRU modulates inflammatory signaling and cardiomyocyte pyroptosis after MI

**Figure 5:**
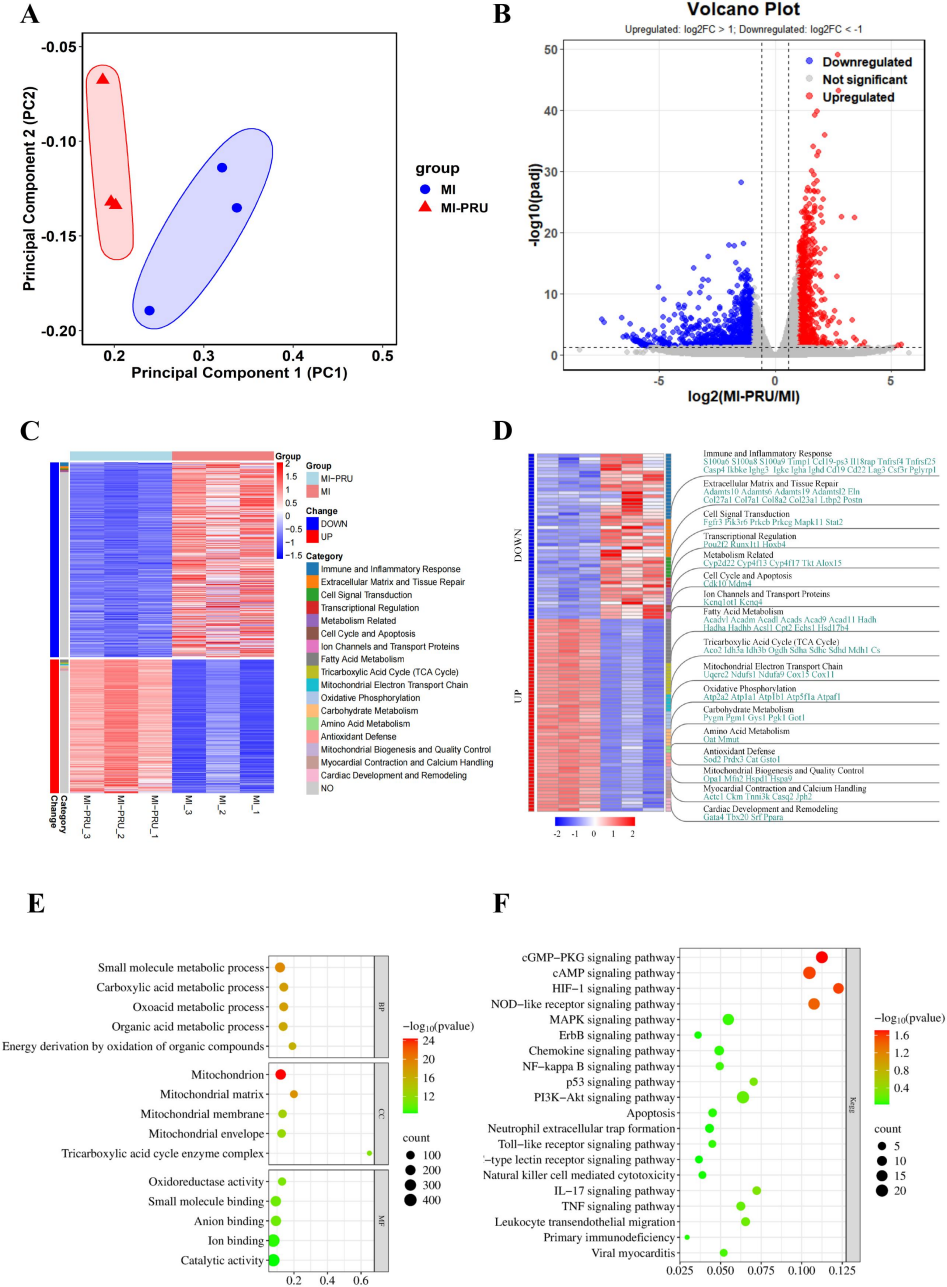
RNA-Seq analysis of PRU-regulated MI mice. **(A)** Component analysis chart. **(B)** Volcano plot of differential genes. **(C)** Heatmap of differential genes. **(D)** Gene enrichment analysis. **(E,F)** GO and KEGG analysis of differential genes.

To investigate the effect of PRU on cardiac gene expression after MI, RNA-seq was performed on heart tissues from the MI and MI + PRU groups. Principal component analysis (PCA) showed a clear separation between the two groups along the PC1 axis, indicating that PRU induced global transcriptomic changes in post-MI cardiac tissues (Figure 5A). Differential expression analysis identified 646 significantly upregulated genes and 944 significantly downregulated genes in the MI + PRU group compared with the MI group, based on the thresholds of |log_2_FC| > 1 and *P* < 0.05 (Figure 5B). Heatmap analysis showed distinct DEG expression patterns with good intragroup consistency, further supporting the PCA results (Figure 5C). Gene set enrichment analysis (GSEA) revealed that the downregulated genes were significantly enriched in biological processes related to immune and inflammatory responses, extracellular matrix remodeling, and tissue repair (Figure 5D). Given the central role of inflammation in MI pathogenesis, GO and KEGG enrichment analyses were further performed on the DEGs. The results showed that these genes were mainly enriched in pyroptosis-related inflammatory pathways (Figures 5E,F). Collectively, these findings suggest that PRU may exert cardioprotective effects after MI by disrupting the interaction between inflammatory signaling and cardiomyocyte pyroptosis.

### 3.5 PRU ameliorates cardiomyocyte pyroptosis

**Figure 6:**
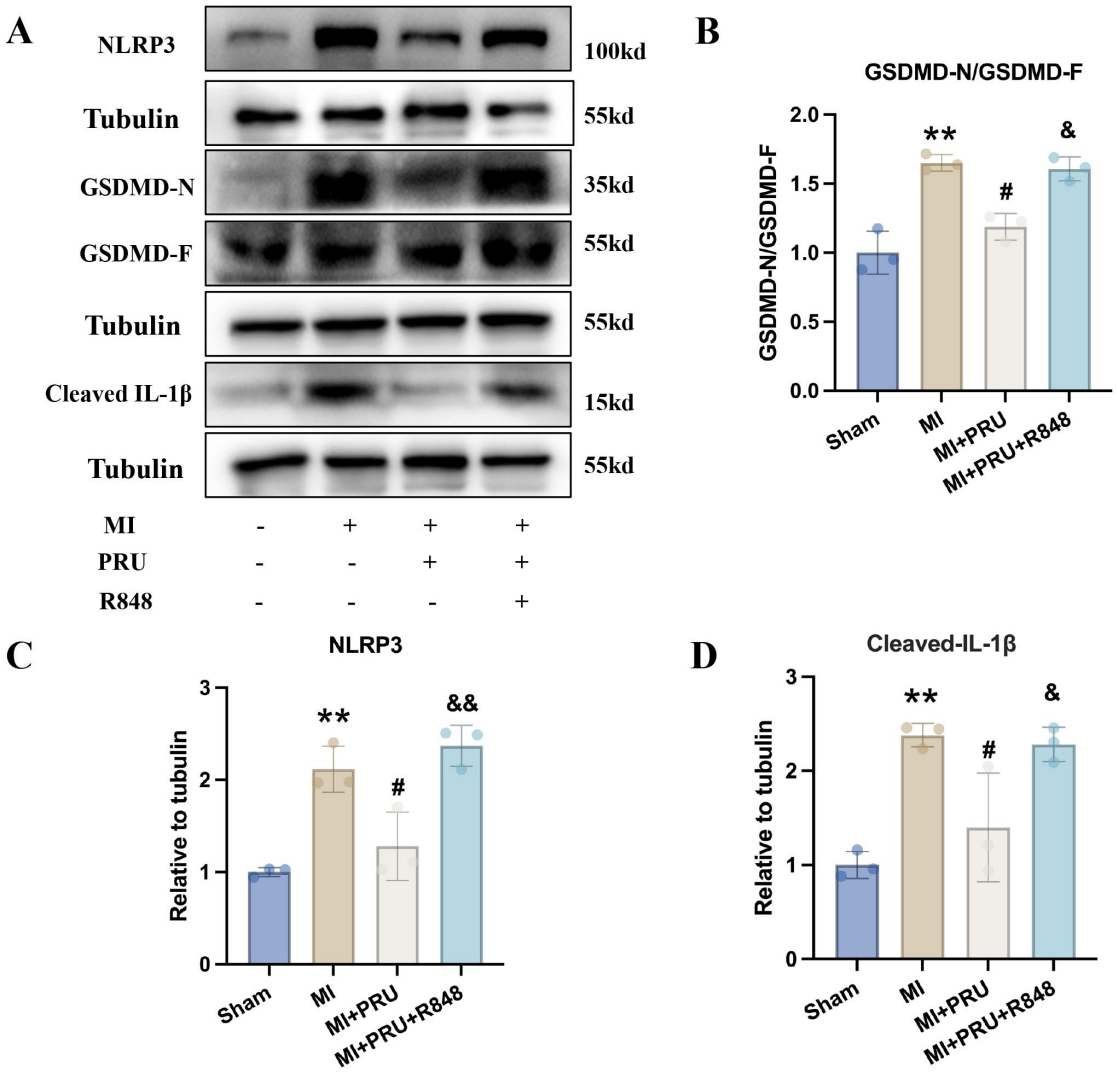
PRU inhibits cardiomyocyte pyroptosis. **(A–D)** Expression of NLRP3, Cleaved IL-1β, and GSDMD-N/GSDMD-F proteins in cardiac tissues measured and quantified by WB, *n* = 3. Data are presented as mean ± SD from independent biological replicates. For panels **(B,D)**, which passed the normality assumption, one-way ANOVA followed by Tukey's *post-hoc* test was applied. For panel **(C)**, which violated the normality assumption, the Kruskal–Wallis test followed by Dunn's *post-hoc* test was used for multiple comparisons. Compared with the Sham group, **P < 0.01, *P < 0.05; compared with the MI group, **^#^***P* < 0.05, **^##^***P* < 0.01; compared with the MI + PRU group, **^&&^***P* < 0.01, **^&^***P* < 0.05.

RNA-seq analysis of cardiac tissues showed that DEGs in PRU-treated MI mice were significantly enriched in inflammation-related pathways, including the TNF, MAPK, and NOD-like receptor signaling pathways. These pathways are closely involved in inflammatory responses and pyroptosis (25–27). To further validate this regulatory effect, we examined the expression of pyroptosis-associated proteins in cardiac tissues. Compared with the sham group, the MI group showed markedly increased protein levels of NLRP3, GSDMD-N, and cleaved IL-1β, indicating abnormal activation of the pyroptosis pathway after MI. In contrast, PRU treatment significantly reduced the expression of these pyroptosis-related proteins, suggesting that PRU effectively suppresses MI-induced pyroptosis activation *in vivo* (Figures 6A–D). Furthermore, *in vitro* experiments with H9C2 cells further demonstrated that PRU exerts a similar regulatory effect on pyroptosis-related proteins in cardiomyocytes (Supplementary Figure S3).

### 3.6 PRU exerts a protective effect against OGD-induced injury in cardiac organoids

**Figure 7:**
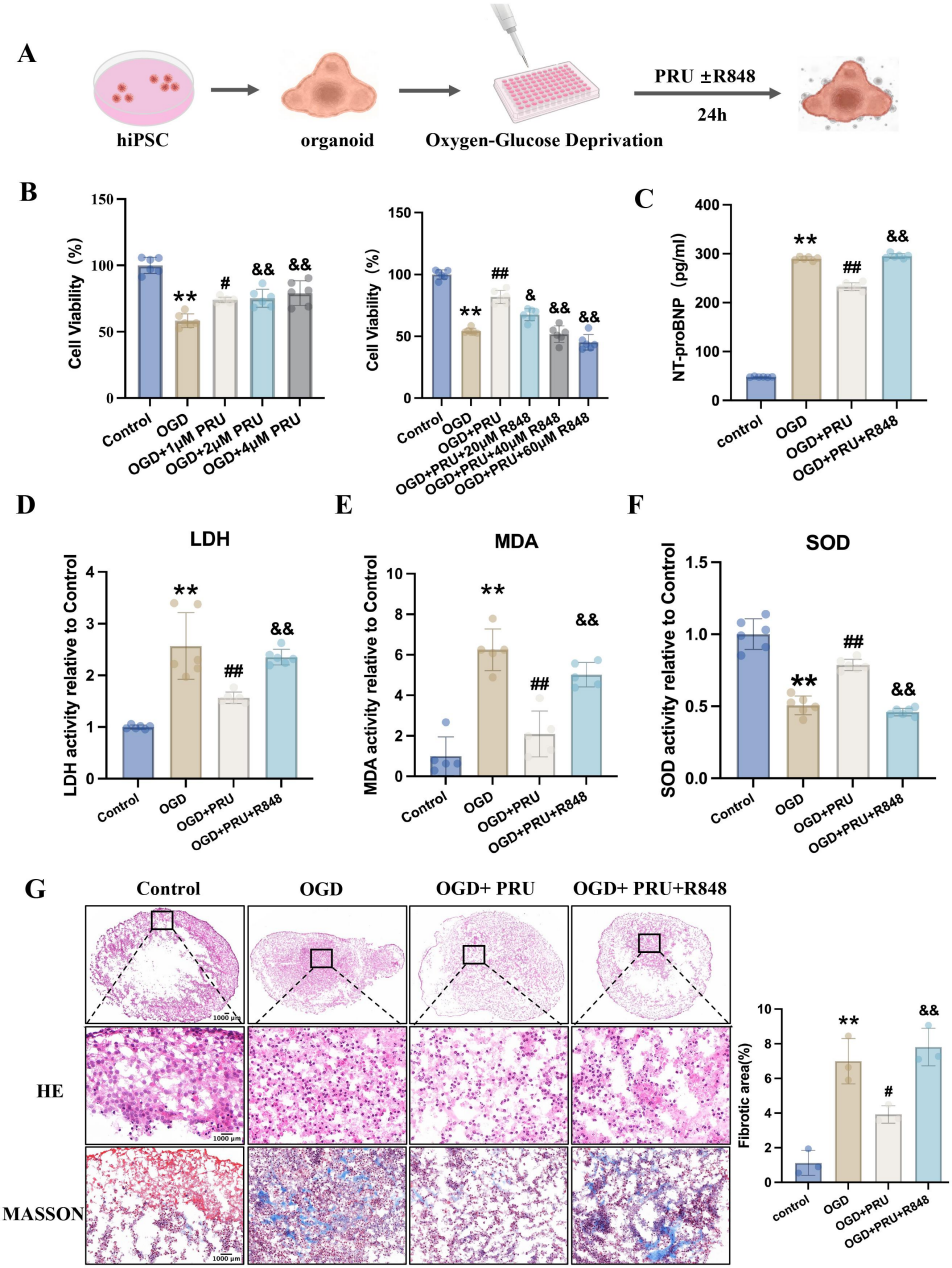
PRU ameliorates OGD-induced injury in cardiac organoids by inhibiting TNF-α. **(A)** Schematic diagram of the experimental timeline for cardiac organoid treatment. **(B)** Viability of cardiac organoids in response to treatment with PRU and R848, *n* = 6. **(C)** Concentrations of NT-proBNP in cardiac organoid culture supernatants measured by ELISA, *n* = 6. **(D–F)** Levels of LDH, MDA and SOD in the supernatant of cardiac organoids, *n* = 6. **(G)** Representative images of cardiac organoids stained with HE and Masson, along with quantitative analysis of cardiac fibrosis area (%), scale bar = 1,000 µm, *n* = 3. Data are presented as mean ± SD from independent biological replicates. For panels **(B,C,F,G)**, which passed the normality assumption, one-way ANOVA followed by Tukey's *post-hoc* test was applied. For panels **(D,E)**, which violated the normality assumption, the Kruskal–Wallis test followed by Dunn's *post-hoc* test was used for multiple comparisons. Compared with the Control group, **P < 0.01, *P < 0.05; compared with the OGD group, **^##^***P* < 0.01, **^#^***P* < 0.05; compared with the OGD + PRU group, **^&&^***P* < 0.01, **^&^***P* < 0.05.

To evaluate the protective effect of PRU *in vitro*, mature cardiac organoids were subjected to OGD for 24 h to establish an OGD-induced injury model (Figure 7A). The optimal concentrations of PRU and R848 in cardiac organoids were determined by CCK-8 assay as 4 μM and 40 μM (Figure 7B). PRU treatment significantly inhibited OGD-induced NT-proBNP release, whereas this effect was markedly reversed by R848 co-treatment, suggesting that PRU can inhibit OGD injury-induced NT-proBNP release (Figure 7C). Consistent with the *in vivo* findings, PRU also ameliorated oxidative stress and cellular injury and reduced fibrosis in cardiac organoids. These protective effects were abolished by R848 (Figures 7D–G), indicating that R848 antagonizes the cardioprotective effects of PRU.

**Figure 8:**
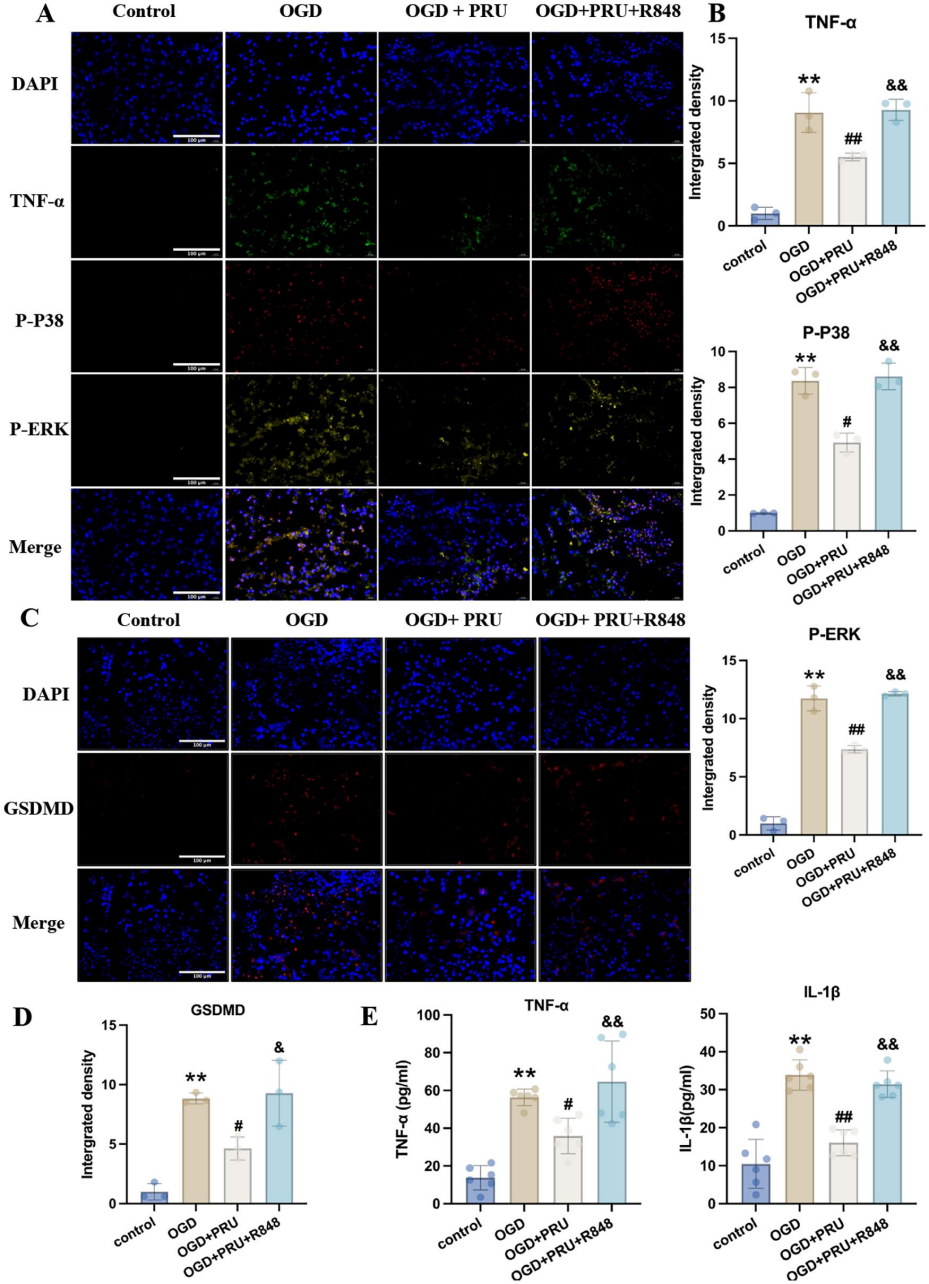
Mechanism underlying the protective effect of PRU against OGD-induced injury in cardiac organoids. **(A,B)** Expression and quantitative analysis of TNF-α, p-p38, and p-ERK in cardiac organoids, scale bar = 100 µm, *n* = 3. **(C,D)** Expression and quantitative analysis of GSDMD in cardiac organoids, scale bar = 100 µm, *n* = 3. **(E)** The levels of TNF-α and IL-1β in the supernatant of cardiac organoids were determined by ELISA, *n* = 6. Data are presented as mean ± SD from independent biological replicates. For panels **(B,E)**, which passed the normality assumption, one-way ANOVA followed by Tukey's *post-hoc* test was applied. For panel **(D)**, which violated the normality assumption, the Kruskal–Wallis test followed by Dunn's *post-hoc* test was used for multiple comparisons. Compared with the Control group, **P < 0.01, *P < 0.05; compared with the OGD group, **^##^***P* < 0.01, **^#^***P* < 0.05; compared with the OGD + PRU group, **^&&^***P* < 0.01, **^&^***P* < 0.05.

Moreover, the immunofluorescence results indicate that PRU markedly reduced TNF-α expression, decreased the p-p38 and p-ERK (Figures 8A,B), and downregulated the pyroptosis-associated protein GSDMD in OGD-exposed cardiac organoids (Figures 8C,D). In contrast, R848 exerted opposite effects on these proteins and significantly reversed the inhibitory effects of PRU on TNF-α and IL-1β release in the supernatant of OGD-treated cardiac organoids (Figure 8E). These *in vitro* findings further demonstrate that PRU protects cardiac organoids against OGD-induced injury by modulating the TNF-α/p38 MAPK/ERK signaling pathway, suppressing cardiomyocyte pyroptosis, and reducing inflammatory cytokine secretion.

## 4 Discussion

This study investigated the cardioprotective effects and underlying mechanisms of PRU, a naturally occurring plant-derived isoflavone, using *in vivo* and *in vitro* models, as well as cardiac organoid technology. Our findings demonstrate that PRU attenuates post-MI myocardial injury by targeting the TNF-α/p38 MAPK/ERK signaling pathway, thereby suppressing inflammatory responses and cardiomyocyte pyroptosis. These effects ultimately improve cardiac function after MI.

Network pharmacology analysis identified TNF-α as a potential core target mediating the cardioprotective effects of PRU. RNA-seq analysis further suggested that PRU may alleviate post-MI injury by regulating downstream inflammatory pathways, particularly the p38 MAPK/ERK signaling cascade. As a central mediator in the pro-inflammatory cytokine network, TNF-α exacerbates myocardial injury by activating MAPK signaling and promoting the release of inflammatory cytokines (12). Consistently, our *in vivo* experiments showed that PRU markedly inhibited MI-induced TNF-α upregulation and reduced the phosphorylation of p38 and ERK. These changes were accompanied by decreased mRNA levels of TNF-α, IL-1β, and IL-6 in cardiac tissues. Importantly, induction of TNF-α expression by R848 reversed the cardioprotective effects of PRU, further supporting that PRU exerts its anti-inflammatory effects primarily through the TNF-α/p38 MAPK/ERK pathway.

After clarifying the upstream signaling events, we further examined the downstream effects of PRU on inflammatory cell death. Given the close relationship between inflammation and pyroptosis, and the enrichment of inflammation-related pathways revealed by RNA-seq, we focused on key pyroptosis-associated molecules. The results showed that PRU significantly suppressed MI-induced upregulation of NLRP3, GSDMD-N, and cleaved IL-1β in murine cardiac tissues. Under physiological conditions, NLRP3 expression in cardiomyocytes remains relatively low, contributing to cardiac immune homeostasis (28, 29). After MI, however, damage-associated molecular patterns released from necrotic cells, together with hypoxia and excessive reactive oxygen species, provide strong activating signals for the NLRP3 inflammasome (30).

The MAPK signaling cascade has also been implicated in NLRP3 inflammasome assembly and pyroptosis activation (25, 31). As a member of the MAPK family, p38 MAPK not only regulates the transcription of NLRP3 and pro-IL-1β by phosphorylating the transcription factor AP-1 (32), but its downstream substrate MK2 also modulates the stability of IL-1β mRNA by phosphorylating proteins such as TTP and hnRNPA0, thereby further amplifying inflammatory signaling at the post-transcriptional level (33, 34). Furthermore, studies have shown that in human primary monocytes, transient LPS stimulation can induce ERK phosphorylation, which in turn mediates ASC clustering and Caspase-1 activation, ultimately promoting the secretion of inflammatory cytokines such as IL-18 (35). These findings suggest a link between ERK phosphorylation status and the sensitivity of the NLRP3 inflammasome to subsequent stimuli. Based on the aforementioned multilevel regulation at the transcriptional, post-transcriptional, and post-translational levels, the upregulation of NLRP3 expression and the activation of caspase-1 ultimately lead to the cleavage of GSDMD. Consequently, the resulting N-terminal pore-forming domain not only triggers pyroptosis but also facilitates the extracellular release of IL-1β and IL-18 (14). In summary, we propose that PRU may inhibit the activation of the NLRP3 inflammasome by suppressing the TNF-α/p38 MAPK/ERK signaling pathway, thereby reducing the release of inflammatory cytokines and ultimately limiting pyroptosis in cardiomyocytes. This mechanism was further validated by our *in vivo* and *in vitro* experiments.

Although PRU has demonstrated significant anti-inflammatory and protective effects in both *in vivo* and *in vitro* settings, its actual bioavailability and efficacy in humans may still be influenced by complex metabolic networks and the tissue microenvironment. In this study, a dose of 40 μg/kg of PRU provided the greatest cardioprotective effect in mice, which translates to a human equivalent dose of approximately 3.2 μg/kg for a 70 kg adult. However, achieving this equivalent dose through daily dietary intake is challenging, given the marked variability of PRU content across food sources. Nevertheless, compared to traditional synthetic anti-inflammatory small molecules, PRU—as a natural isoflavone compound—offers multi-target regulation and a broader therapeutic window, advantages that mitigate compensatory resistance and systemic toxicity, making it suitable for long-term intervention via nutritional fortification or low-dose dietary supplementation. Future studies should systematically characterize its ADME (Absorption, Distribution, Metabolism, and Excretion) profile in large animal and preclinical models, and enhance its bioavailability and target-organ accumulation through formulation optimization or delivery systems, thereby establishing a pharmacokinetic and safety profile to support clinical translation.

This study has several limitations. First, there may be differences between primary rodent cardiomyocytes and cardiac organoids derived from hiPSCs in terms of electrophysiological properties, metabolic dependence, and maturity, which may affect their responses to stress and therapeutic interventions. Cardiac organoids, owing to their multicellular nature, may better reflect human-specific cellular behavior and disease-related pathways. However, neither the H9C2 cell line nor cardiac organoids can replace primary cardiomyocytes as the classic *in vitro* model in cardiovascular research. Therefore, future studies should systematically evaluate the cardioprotective effects of PRU, as well as its anti-inflammatory and anti-pyroptotic molecular mechanisms, in a primary cardiomyocyte model of OGD-induced injury. This is crucial for validating our findings, assessing species-specific differences, and strengthening the translational medical significance of this study. Second, while this study primarily focuses on the regulatory role of PRU in pathological transcriptional changes following myocardial infarction, it cannot rule out the possibility of direct effects resulting from its status as a natural small molecule; this requires further investigation.

## 5 Conclusion

In summary, this study reveals a novel mechanism by which the food-derived isoflavone PRU ameliorates post-MI myocardial injury. PRU targets TNF-α and inhibits the p38 MAPK/ERK signaling pathway, thereby reducing inflammatory responses and cardiomyocyte pyroptosis. These findings expand the potential application of natural dietary compounds in cardiovascular protection and provide a pharmacological basis for the development of PRU as a functional food-derived candidate for adjunctive MI therapy.

## Data Availability

The original contributions presented in the study are included in the article/Supplementary Material, further inquiries can be directed to the corresponding author.

## Supplementary material

Supplementary Figure S1 **Screening of related targets and validation of MI model. (A)** Validation of related targets underlying PRU-mediated attenuation of OGD-induced injury in H9C2 cardiomyocytes by qPCR, n=3. **(B)** TTC staining and quantitative analysis of cardiac tissues at 48 h post-MI, n=5. **(C)** Statistical chart of mouse echocardiography LVEF and LVFS 48 h after MI surgery. Data are presented as mean ± SD from independent biological replicates. For panel (B), which passed the normality assumption, Student's t-test was applied. For panels (A, C), which passed the normality assumption, one-way ANOVA followed by Tukey's post-hoc test was applied. Note: Compared with the Control or Sham group, ***P* < 0.01, **P* < 0.05; compared with the OGD or MI group, ^**#****#**^*P* < 0.01, ^**#**^*P* < 0.05.

Supplementary Figure S2 **PRU alleviates cardiomyocyte injury by inhibiting the TNF-α/p38 MAPK/ERK signaling pathway. (A-B)** Screening of optimal concentrations of PRU and R848 by CCK-8 assay, n=4. **(C)** Effects of different concentrations of PRU on LDH levels in the supernatant of cardiomyocytes, n=6. **(D-E)** Expression of TNF-α, p-p38, and p-ERK proteins in OGD-induced injured cardiomyocytes measured and quantified by WB, n=3. Data are presented as mean±SD from independent biological replicates. For panels (A-C, E), which passed the normality assumption, one-way ANOVA followed by Tukey's post-hoc test was applied. Note: Compared with the Control group, ***P* < 0.01, **P* < 0.05; compared with the OGD group, ^**#****#**^*P* < 0.01, ^**#**^*P* < 0.05; compared with the OGD + PRU group, ^**&****&**^*P* < 0.01, ^**&**^*P* < 0.05.

Supplementary Figure S3 **PRU alleviates OGD-induced pyroptosis in cardiomyocytes. (A-D)** Expression of NLRP3, Cleaved IL-1β, and GSDMD-N/GSDMD-F proteins in OGD-induced injured cardiomyocytes measured and quantified by WB, n=3. Data are presented as mean±SD from independent biological replicates. For panels (B-D), which passed the normality assumption, one-way ANOVA followed by Tukey's post-hoc test was applied. Note: Compared with the Control group, ***P* < 0.01, **P* < 0.05; compared with the OGD group, ^**#****#**^*P* < 0.01, ^**#**^*P* < 0.05; compared with the OGD + PRU group, ^**&****&**^*P* < 0.01, ^**&**^*P* < 0.05.
